# Case Report: Long-Term Chemotherapy With Hydroxyurea and Prednisolone in a Cat With a Meningioma: Correlation of FDG Uptake and Tumor Grade Assessed by Histopathology and Expression of Ki-67 and p53

**DOI:** 10.3389/fvets.2021.576839

**Published:** 2021-01-26

**Authors:** Taesik Yun, Yoonhoi Koo, Hakhyun Kim, Wonguk Lee, Soochong Kim, Dong-In Jung, Mhan-Pyo Yang, Byeong-Teck Kang

**Affiliations:** ^1^Laboratory of Veterinary Internal Medicine, College of Veterinary Medicine, Chungbuk National University, Cheongju, South Korea; ^2^Department of Nuclear Medicine, Chungbuk National University Hospital, Cheongju, South Korea; ^3^Department of Veterinary Medicine, College of Veterinary Medicine, Chungbuk National University, Cheongju, South Korea; ^4^Institute of Animal Medicine, College of Veterinary Medicine, Gyeongsang National University, Jinju, South Korea

**Keywords:** brain tumor, chemotherapy, feline, hydroxyurea, positron emission tomography

## Abstract

A 15.5-year-old, neutered, male, domestic shorthair cat was presented with neurologic dysfunctions. At presentation, an obtunded mental status and vestibular ataxia were identified. On neurologic examination, postural reactions were decreased-to-absent in all four limbs, and pupillary light reflexes showed bilaterally delayed results. Magnetic resonance imaging was performed, and a demarcated lesion was identified in the third ventricle. The cat was tentatively diagnosed with a brain tumor, which was suspected to be a meningioma. The cat was treated with hydroxyurea and prednisolone. Mental status was considered more alert, and ataxia improved following treatment. On the 106th day after the commencement of treatment, a ^18^F-fluorodeoxyglucose (FDG)-positron emission tomography (PET) scan was performed. On the PET images, a hypermetabolic region was found in the lesion. The average standardized uptake value of FDG was 2.47, and the tumor-to-normal-tissue ratio was 1.25. The cat died 408 days following the commencement of treatment, and a grade 1 meningioma was confirmed by postmortem histopathology. Immunohistochemistry for Ki-67 and p53 was performed. The labeling indices of Ki-67 and p53 were 2.56 and 0%, respectively. This case shows that chemotherapy with hydroxyurea and prednisolone may be considered in the treatment of feline meningiomas. Furthermore, this is the first case describing the application of FDG-PET to visualize a naturally occurring meningioma in a cat.

## Introduction

Meningiomas account for 58.1% of the CNS neoplasms and are the most common intracranial primary tumors in cats (1). They are generally benign, slowly progressing, mostly solitary, and originate from the meninges (arachnoid cells). They occur mainly in elderly cats (older than 10 years of age) (1–3). Male cats are affected more than females (1–3). In cats, the common locations of meningiomas are known to be the supratentorial meninges, including the third ventricle, parietal, temporal, and frontal lobes (1). Multiple meningiomas are common, accounting for 14.7–17.2% of all meningioma cases in cats (1, 4). Approximately 21.5% of the cats with confirmed meningiomas show no specific neurologic signs (1). There are several treatment options, including palliative therapy, surgery, radiotherapy, chemotherapy, and combination therapies, to treat feline meningiomas (1, 5, 6). In human oncology, positron emission tomography (PET) has been frequently used to differentiate between benign and malignant meningiomas (7, 8), and in the identification of metastases (9) and residual tumors following treatment (10). The representative radiopharmaceutical tracer used in PET is ^18^F-fluorodeoxyglucose (FDG)—glucose molecule labeled with ^18^F radioisotope. FDG uptake correlates proportionally with the level of glucose metabolism in tissues; thus, FDG-PET is generally used in oncology to identify tumors (7–10). In veterinary medicine, some FDG-PET characteristics of feline malignancy cases have been reported (11, 12), but there are no reports of FDG-PET findings in feline brain tumors. This is the first reported case describing the application of FDG-PET to identify a naturally occurring meningioma in a cat.

## Case Presentation

A 15.5-year-old, neutered, male, domestic shorthair cat with neurologic dysfunctions was presented to our institution. An initial clinical symptom of mildly obtunded mental status, that began 168 days before presentation, was noted. However, before presentation, the cat had shown acute deterioration of neurologic signs, including exacerbation of mental status and vestibular ataxia, falling toward the right side. On physical examination, the cat weighed 4.96 kg, had a pulse rate of 203 beats per minute, a respiratory rate of 45 breaths per minute, a rectal temperature of 38.0°C, and a systolic blood pressure of 140 mmHg. A complete blood cell count, chemistry profile, electrolyte, and blood-gas tests revealed normal results. On neurologic examination, postural reactions were decreased-to-absent in all four limbs, and cranial nerve examination findings were normal, except for bilaterally delayed direct and consensual pupillary light reflexes. Based on the obtunded mental status, vestibular ataxia, and delayed pupillary light reflexes, the neuroanatomical localization was to the forebrain and brainstem.

**Figure 1:**
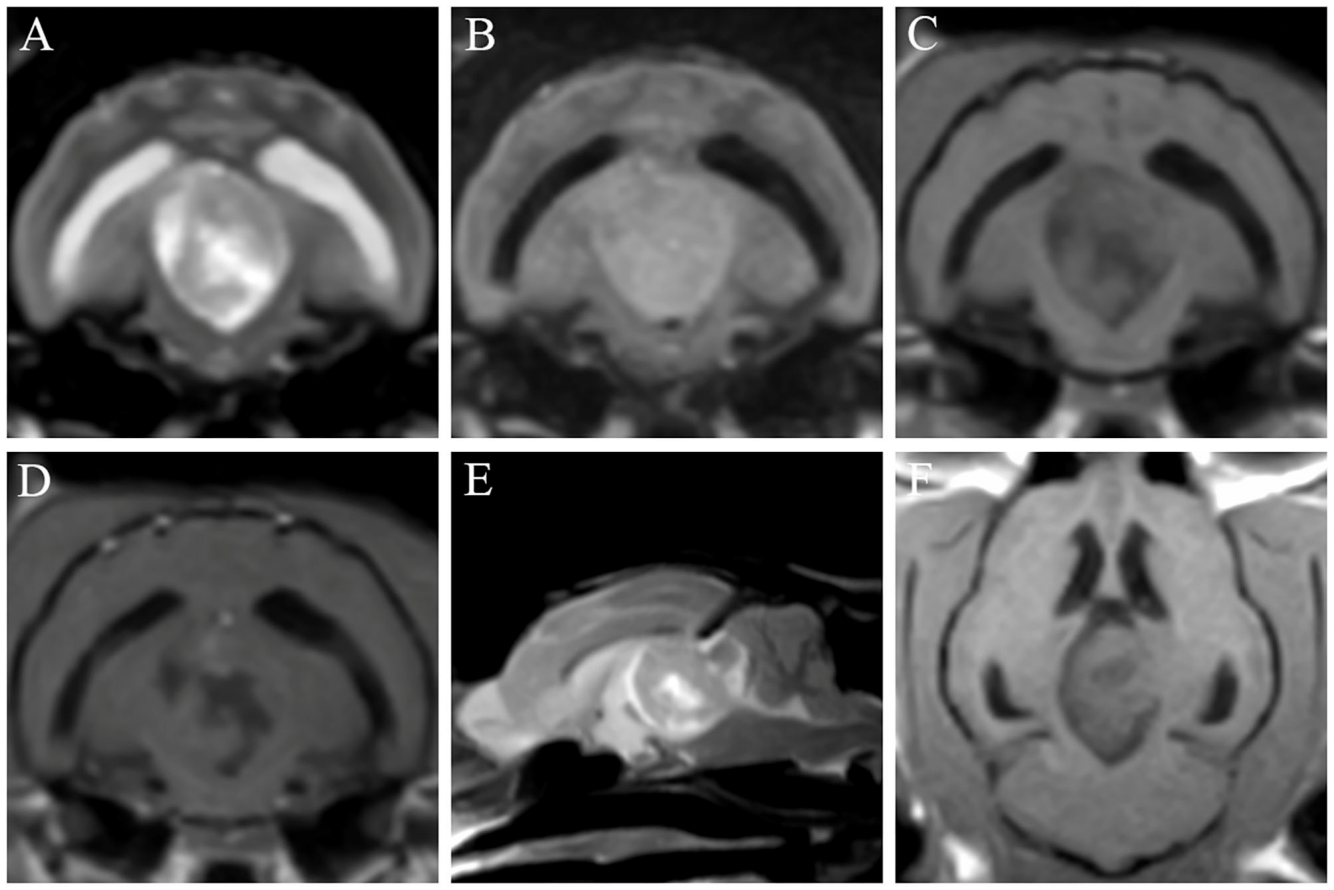
Findings of the first magnetic resonance imaging of a cat with a meningioma. T2-weighted **(A,E)**, fluid-attenuated inversion recovery (FLAIR) **(B)**, T1-weighted **(C,F)**, and post-contrast **(D)** images were obtained. An oval-shaped mass is identified in the third ventricle, showing mass effects on the rostral cerebellum, brainstem, and occipital cortex. The mass appears hypointense-to-isointense in T1-weighted images **(C,F)** and isointense-to-hyperintense in T2-weighted **(A,E)** and FLAIR **(B)** images. The lesion is heterogeneously enhanced in the post-contrast image **(D)**.

Magnetic resonance imaging (MRI) examination of the brain was performed using a 0.3-Tesla unit (Airis II, Hitachi, Tokyo, Japan). General anesthesia was induced with intravenous administration of propofol (6 mg/kg; Provive, Myungmoon Pharm. Co., Ltd., Seoul, South Korea) and maintained by inhalation of 2.0% isoflurane (Terrell, Piramal Critical Care, Bethlehem, PA, USA) with a circle rebreathing circuit. T1-weighted (pre- and post-contrast), T2-weighted, and fluid-attenuated inversion recovery (FLAIR) images were obtained in the transverse, sagittal, and dorsal planes. An oval-shaped extra-axial mass (length × height × width: 2.0 × 1.7 × 1.4 cm) was identified in the third ventricle, compressing the rostral cerebellum, brainstem, and the occipital cortex (Figure 1). It was well-demarcated with an isointense-to-hyperintense signal, relative to the surrounding parenchyma, in T2-weighted (Figures 1A,E) and FLAIR (Figure 1B) images, and with a hypointense-to-isointense signal, relative to the surrounding parenchyma, in T1-weighted images (Figures 1C,F). After administration of gadolinium-diethylenetriamine pentaacetic acid [0.1 mmol/kg, IV; Omniscan^TM^, GE Healthcare (Shanghai), Co., Ltd., China], post-contrast images were taken and the oval-shaped mass lesion appeared heterogeneously enhanced (Figure 1D). A cerebrospinal fluid tap was not performed due to suspicion of increased intracranial pressure and transtentorial and foramen magnum herniation visible on MRI. Based on the patient's history, symptoms, clinical signs, and MRI features, a brain tumor was tentatively diagnosed. Specifically, a meningioma was strongly suspected; however, other tumors, including ependymoma, choroid plexus tumors, lymphoma, and histiocytic sarcoma, needed to be considered.

**Figure 2:**
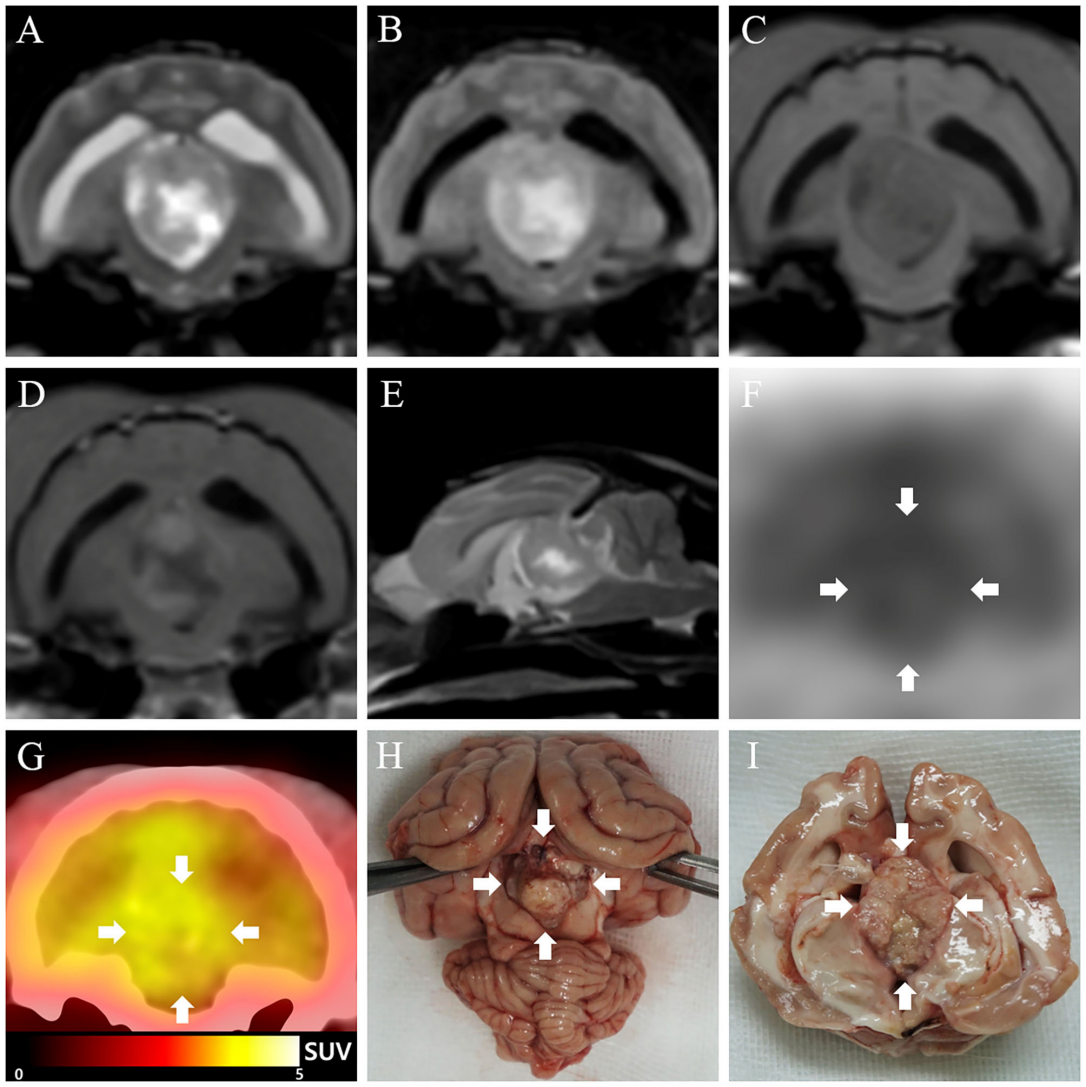
Second magnetic resonance imaging (MRI), ^18^F-fluorodeoxyglucose (FDG)-positron emission tomography (PET), and necropsy findings of meningioma in a cat. **(A–E)** On the second MRI (106 days after the commencement of treatment), the size of the mass appears similar to that measured at the first MRI. **(F)** On FDG-PET image, high FDG uptake is seen, represented with a black color, while low FDG uptake is represented with a white color. The FDG uptake in the tumor lesion (arrows) is relatively higher than that of the surrounding regions. **(G)** On PET/computed tomography fusion image, high FDG uptake is represented as yellow color, while low FDG uptake is represented by black to red color. Glucose metabolism in the third ventricle (arrows) is relatively higher than that in the surrounding regions. **(H,I)** A well-demarcated, oval-shaped mass (arrows) is observed in the third ventricle.

The cat was initially treated with oral prednisolone (0.5 mg/kg twice daily; Solondo®, Yuhan, Seoul, South Korea) as a palliative therapy to decrease the intracranial pressure by relieving the tumor-associated brain edema (13) and decreasing cerebrospinal fluid production (14). A week after the palliative therapy, signs of neurologic recovery were observed, except for a persistent mildly obtunded mental status. Based on the results of a follow-up examination on day 42 after the commencement of palliative therapy, chemotherapy using hydroxyurea (25 mg/kg, PO; Hydrin®, Korea United Pharm., Seoul, South Korea) was started. Hydroxyurea was prescribed once daily for the first 2 weeks and then tapered to once every alternate day. Prednisolone administration, with dosage ranging from 0.5 mg/kg once daily to 1 mg/kg twice daily, was maintained throughout the chemotherapy based on clinical signs, such as mental status and severity of ataxia. On the 106th day (64 days after the chemotherapy) after the commencement of treatment, a second MRI was performed to evaluate therapeutic effectiveness by identifying the tumor size. The second MRI showed a tumor size similar to that seen in the first MRI (length × height × width: 2.0 × 1.6 × 1.3 cm) (Figures 2A–E). Furthermore, immediately after the second MRI, we performed an FDG-PET scan because it could identify malignancy and tumor metastasis, and also because the FDG-PET findings of feline meningiomas have not been previously reported. FDG (5.92 MBq/kg) was administered intravenously into a saphenous vein, followed by flushing of residual FDG with 5 ml of 0.9% normal saline. Low-dose computed tomography (CT) images were acquired prior to the PET scan. Twenty-minute PET scans were obtained 1 h after FDG injection. PET image analysis was conducted using OsiriX MD v10.0 (Pixmeo Sarl, Geneva, Switzerland). The regions of interest (ROIs) were drawn manually on the PET/CT fusion images. Metabolic activity inside the ROIs was converted to a standardized uptake value (SUV) as follows:

SUV = average tissue concentration of FDG (MBq/ml)/injected dose (MBq) per body weight (g)

For more objective evaluation of the metabolic activity, the tumor-to-normal-tissue (T/N) ratio was generated by dividing the tumor SUV by the SUV of the normal tissue (cerebral cortices of the frontal, parietal, and temporal lobes). On visual evaluation of the PET images, a hypermetabolic region was found in the tumor lesion (Figures 2F,G). The mean and maximal SUVs of the tumor were 2.47 and 2.84, respectively, and the T/N ratio was 1.25. Abnormal uptake of FDG was not observed in any other organs except the brain. A complete blood cell count, chemistry profile, electrolyte, and blood-gas tests were periodically performed (about once a month) to identify adverse effects of hydroxyurea and prednisolone. Mild anemia (packed cell volume, 25–30%; reference range, 30.3–52.3%) occurred right after the commencement of chemotherapy and persisted throughout the treatment period, but without further deterioration. Adverse effects other than anemia were not observed. The cat showed clinical deteriorations, including obtunded mental status and vestibular ataxia, on days 66, 221, and 320; the dose of prednisolone was thus adjusted based on worsening of clinical signs, while the hydroxyurea dosage was maintained. Mental status became more alert and ataxia improved whenever the dose of prednisolone increased; however, the cat died on the 408th day after the commencement of treatment (365 days after the commencement of chemotherapy).

**Figure 3:**
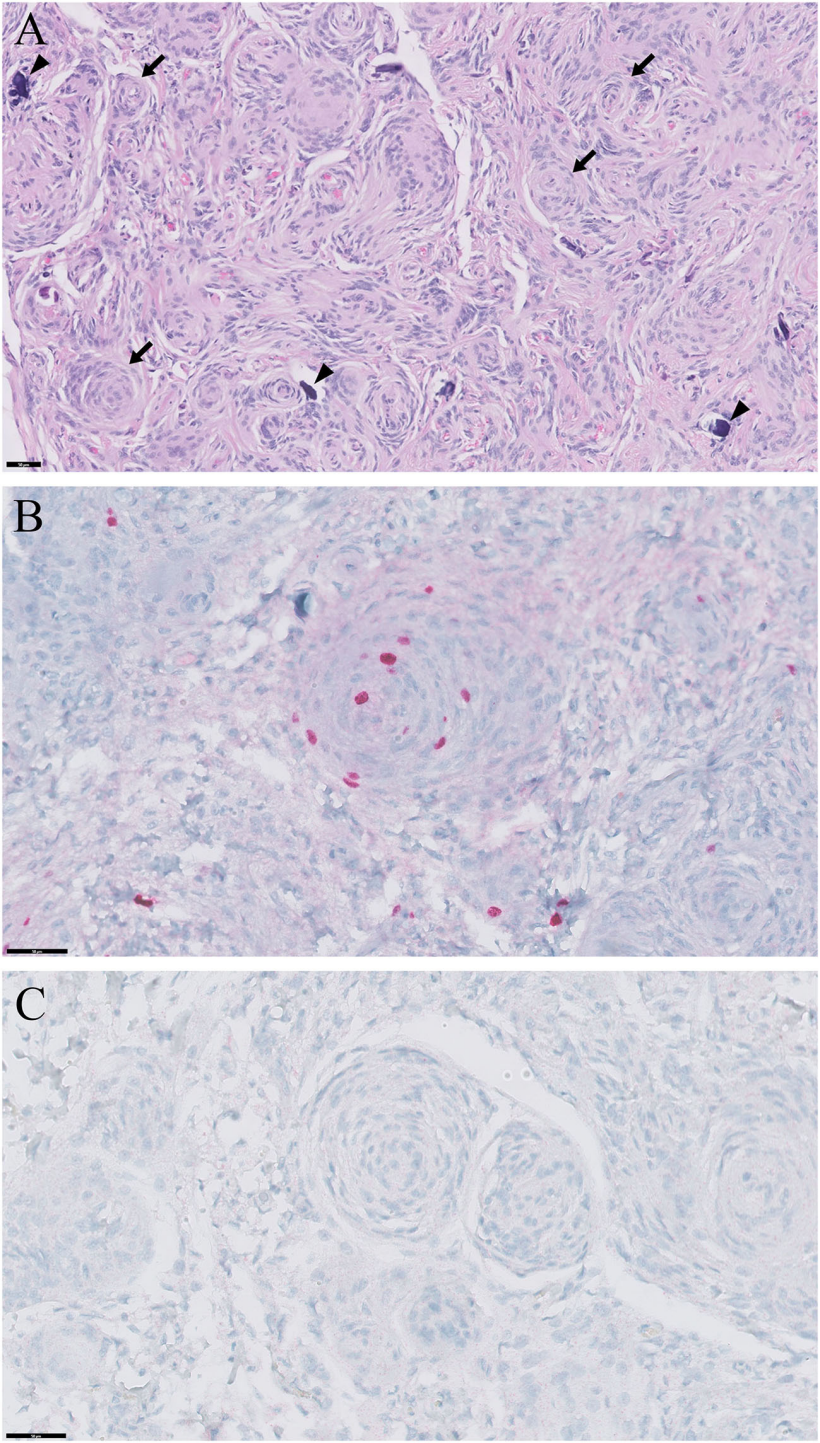
Histopathological and immunohistochemical findings of meningioma in a cat. **(A)** Extensive concentric whorls (arrows) of polygonal to oblong cells, and spindle shaped cells with interlacing bundles of fibroblast-like cells are observed. Some psammoma bodies (mineralized concretions; arrowheads) are scattered throughout the mass. Hematoxylin and eosin; ×200 magnification; bar = 50 μm. **(B)** Positive immunoreactivity to Ki-67. Hematoxylin and eosin; ×400 magnification; bar = 50 μm. **(C)** Negative immunoreactivity to p53. Hematoxylin and eosin; ×400 magnification; bar = 50 μm.

At necropsy, a well-demarcated, oval-shaped mass was found in the third ventricle (Figures 2H,I). Histopathology of the mass showed extensive concentric whorls of cells, arranged in a polygonal to oblong pattern, and spindle-shaped fibroblast-like cells with interlacing bundles (Figure 3A). Some psammoma bodies (mineralized concretions) were scattered throughout the mass. Based on these histopathological features and the World Health Organization classification of human meningiomas (15), the current case was definitively diagnosed as a grade 1 meningioma. Immunohistochemical staining for Ki-67 (proliferation index) and p53 (index of cell cycle regulation) was also performed. The labeling indices of Ki-67 and p53 were measured as a percentage of Ki-67- and p53-positive cells by counting 1,000 tumor cell nuclei in fields with the highest number of positive cells. The immunohistochemistry showed that the tumor cells were positive for Ki-67 and negative for p53 (Figures 3B,C). The labeling index of Ki-67 was 2.56%.

## Discussion

This is the first reported case describing the application of FDG-PET to detect a naturally occurring meningioma in a cat. Immunohistochemistry for prognostic markers, such as Ki-67 (a proliferation marker) and p53 (index of cell cycle regulation), was performed to assess the tumor and to correlate the findings with FDG-PET results.

FDG-PET has been used for the assessment of glucose metabolism in humans (7–10, 16). Since deranged glucose metabolism is a common feature in many malignant tumors, it is primarily used to identify metabolically active lesions for differentiation of benign and malignant tumors in pancreatic mass, pulmonary nodules, and remnant lesions after chemotherapy in lymphoma (8, 16). FDG-PET has been also used for staging and monitoring therapeutic response of cancer (10, 16), and in the diagnosis of Alzheimer's disease based on reduced cerebral metabolism, specially, in the cortical regions (17). In veterinary medicine, some FDG-PET characteristics of feline malignancy have been reported (11, 12), but there are no reports of FDG-PET findings in feline brain tumors. FDG-PET has been used as an imaging modality to differentiate between low- and high-grade meningiomas in humans (18). The SUV of low-grade (grade I; SUV: 2.35 ± 0.91) meningiomas was lower than that of high-grade (grade II and III; SUV: 4.83 ± 1.86) meningiomas in humans (18). The T/N ratios of low- and high-grade meningiomas were also 0.62 ± 0.18 and 2.33 ± 1.99, respectively, in humans (18). In the present study of feline grade I meningioma, the mean SUV was 2.47 and the T/N ratio was 1.25. Compared with human results (18), the SUV of feline low-grade meningioma was similar to that of human low-grade meningiomas, whereas the T/N ratio of feline low-grade meningioma was higher than that of human low-grade meningiomas, but lower than that of human high-grade meningiomas. Potential reasons for this difference in the T/N ratio between humans and cats include differences in the pathophysiology of meningiomas and also potential treatment protocols and treatment durations, which could lead to tissue disruption and pathophysiological changes. Furthermore, although no significant difference in the T/N ratio was found among patients with and without recurrence of meningioma after surgical resection, univariate analysis identified SUV of tumor lesions as significant predictive factors for meningioma recurrence in humans (19). The T/N ratio was found to correlate significantly with the Ki-67 labeling index (tumor proliferation index) and the mitotic count per 10 high-power fields, showing that the uptake of FDG could reflect the proliferative activity of meningiomas in humans (19). We could not confirm whether the FDG uptake and T/N ratio were related to tumor recurrence, and the proliferative activity could not be assessed due to the small sample size. Therefore, more cases need to be analyzed to establish general diagnostic criteria to differentiate between benign and malignant meningiomas, and to predict the recurrence and proliferative potential of meningiomas in veterinary medicine.

Although grading of meningiomas by histologic evaluation has clinical and prognostic implications, it is hard to predict the outcome of meningiomas. Therefore, to predict the prognosis of meningiomas, several studies have evaluated the use of immunohistochemical markers of meningiomas in humans (20–23). Among the various markers, Ki-67 and p53 are known to be significantly correlated with the grade of meningiomas, affecting the grade and subtype (20–23). For these reasons, we performed immunohistochemistry for Ki-67 and p53 in this study. The presentation of the nuclear protein Ki-67 is used extensively as a proliferation index because it is primarily expressed in proliferating mammalian cells (24). The average Ki-67 labeling index values of grade 1, 2, and 3 meningiomas in humans were reported to be 1.5–3.54, 6.2–11.9, and 10.2–19.5%, respectively (20–22). The Ki-67 labeling index value of feline grade 1 meningioma for our patient was 2.56%; this value corresponds to the Ki-67 value of human grade 1 meningiomas. The transcription factor p53 suppresses tumor growth through the regulation of dozens of target genes with diverse biological functions (25). Mutations in p53 or alterations in its regulatory network are major causes of tumorigenesis in many malignant tumors (25). The p53 labeling index values of human grade 1, 2, and 3 meningiomas were reported to be 0–1.1, 1.6–12.8, and 6.39–15.6%, respectively (20, 21, 23). The p53 labeling index value of our patient with feline grade 1 meningioma was 0%; this value corresponds to the value reported for human grade 1 meningiomas.

There are several treatment options for feline meningiomas, including palliative therapy, chemotherapy, surgery, radiotherapy, and combination therapies (1, 5, 6). Typically, glucocorticoids and anticonvulsive drugs are the mainstay of palliative care. Palliative therapy with glucocorticoids can reduce peritumoral edema by decreasing the vascular permeability (13). Although there have been no meaningful data on the survival time of feline meningiomas with palliative therapy, some clinicians anecdotally recognize that the survival times could be relatively long in patients who receive only palliative therapy. Surgical removal/debulking and radiotherapy have been commonly used to treat meningiomas, with good prognosis and prolonged survival time (1, 5, 26). Surgery has been reported to confer the longest median survival time (22.8–37 months) among the existing treatments for feline meningiomas (1, 2, 26), whereas the median survival time associated with radiotherapy was 365 days (5). Combined therapy with surgery, radiotherapy, and chemotherapy constitute the conventional treatment for brain tumors. Although the data on survival time with combined therapy in feline meningiomas have not been reported to date, the median survival time of combination therapy with surgery and radiation therapy in canine meningiomas was 14.6–30+ months (27–29).

For cats that receive limited surgical and radiation therapy, medical treatment, including administration of chemotherapeutic agents, could be considered as an alternative. The effects of chemotherapy for treating meningiomas have been described in humans and veterinary patients (6, 30–34). Although chemotherapy has shown mixed effects in humans (33, 34), some chemotherapeutic agents have shown beneficial effects in meningiomas when used for long periods in humans and veterinary patients (6, 32, 34). Hydroxyurea is an oral antineoplastic drug that induces tumor cell apoptosis (35) and selectively inhibits ribonucleotide reductase, thereby preventing DNA synthesis (36). In the current case, the survival time was 408 days using hydroxyurea and prednisolone, without surgery and radiotherapy. There were three events of clinical deteriorations (obtunded mental status and vestibular ataxia) at 66, 221, and 320 days after treatment initiation. Whenever these deteriorations occurred, the dose of prednisolone was increased (day 66, from 0.5 mg/kg once daily to 0.5 mg/kg twice daily; day 221, from 0.5 mg/kg twice daily to 1 mg/kg twice daily; day 320, from 0.75 mg/kg twice daily to 1 mg/kg twice daily), while the dose of hydroxyurea was maintained; this regimen resulted in the improvement of neurologic signs. Complete remission was not observed in this case, and the size of mass remained the same at both first and second MRI scans; however, the cat responded to increased dosage of prednisolone whenever clinical deterioration occurred. Therefore, hydroxyurea and prednisolone were thought to have a role in preventing tumor growth and reducing the cerebral edema-associated periodic clinical deterioration, respectively.

To the best of our knowledge, this is the first case report to describe FDG-PET results in a case of feline grade 1 meningioma. The mean SUV and the T/N ratio were 2.47 and 1.25, respectively, and the labeling indices of Ki-67 and p53 were 2.56 and 0%, respectively. In the future, these data may provide valuable diagnostic information to better understand feline meningiomas in veterinary medicine, and further studies are needed to establish diagnostic criteria for feline meningiomas.

## Data Availability Statement

The original contributions presented in the study are included in the article/supplementary material, further inquiries can be directed to the corresponding author/s.

## Ethics Statement

Ethical review and approval was not required for the animal study because the study was a case report, thus no ethical approval was required. Written informed consent was obtained from the owners for the participation of their animals in this study.

## Author Contributions

TY, YK, HK, D-IJ, M-PY, and B-TK contributed management of case. SK was in charge for the histopathological evaluation. WL helped with the PET/CT operation. TY wrote the first draft of the manuscript. TY, HK, SK, D-IJ, M-PY, and B-TK were participated in the revision of the manuscript. All authors read, commented on, and approved the final manuscript.

## Conflict of Interest

The authors declare that the research was conducted in the absence of any commercial or financial relationships that could be construed as a potential conflict of interest.
